# Dielectrophoretic Micro-Organization of Chondrocytes to Regenerate Mechanically Anisotropic Cartilaginous Tissue

**DOI:** 10.3390/mi12091098

**Published:** 2021-09-11

**Authors:** Yoshitaka Takeuchi, Shogo Miyata

**Affiliations:** 1 Graduate School of Science and Technology, Keio University, 3-14-1 Hiyoshi, Kohoku-ku, Yokohama 223-8522, Japan; dai_bamboo77@yahoo.co.jp; 2 Faculty of Science & Technology, Keio University, 3-14-1 Hiyoshi, Kohoku-ku, Yokohama 223-8522, Japan

**Keywords:** dielectrophoresis, chondrocyte, mechanical anisotropy, tissue engineering, cell patterning

## Abstract

Recently, many studies have focused on the repair and regeneration of damaged articular cartilage using tissue engineering. In tissue engineering therapy, cells are cultured in vitro to create a three-dimensional (3-D) tissue designed to replace the damaged cartilage. Although tissue engineering is a useful approach to regenerating cartilage, mechanical anisotropy has not been reconstructed from a cellular organization level. This study aims to create mechanically anisotropic cartilaginous tissue using dielectrophoretic cell patterning and gel-sheet lamination. Bovine chondrocytes were patterned in a hydrogel to form line-array cell clusters via negative dielectrophoresis (DEP). The results indicate that the embedded chondrocytes remained viable and reconstructed cartilaginous tissue along the patterned cell array. Moreover, the agarose gel, in which chondrocytes were patterned, demonstrated mechanical anisotropy. In summary, our DEP cell patterning and gel-sheet lamination techniques would be useful for reconstructing mechanically anisotropic cartilage tissues.

## 1. Introduction

Articular cartilage covers bone ends in diarthrodial joints and is an inhomogeneous, anisotropic, and viscoelastic connective tissues that serve as a low-friction load-bearing material [1,2,3]. Cartilage plays an important role in mammalian skeletal movements. Due to the avascular nature, low cell density, and low proliferative activity of chondrocytes, hyaline cartilage cannot regenerate after injury; wear and tear; or degeneration through common diseases, such as osteoarthritis [4,5]. Therefore, severe cartilage damage often requires surgical treatment. Current clinical approaches to generating new cartilaginous tissue include debridement, microfracture, autologous chondrocyte transplantation, and mosaicplasty. However, it is difficult to regenerate hyaline cartilage using these therapeutic approaches.

Tissue engineering approaches have been developed to restore articular cartilage damage, which involve culturing autologous chondrocytes in vitro to create a three-dimensional (3-D) tissue designed to replace the damaged tissue [6,7,8,9]. In clinical studies, chondrocyte sheets or chondrocyte cultures in atelocollagen gel were transplanted to treat osteochondral defects [6,8]. In native articular cartilage, chondrocyte organization varies with the depth of the articulating surface [10]. Collagen fibers, which are one of the main components of cartilage, align in response to the direction of cyclic deformations induced by daily walking or other activities. Moreover, this alignment is related to cell biosynthesis and tissue anisotropy. However, these inhomogeneous and anisotropic structures of articular cartilage cannot be regenerated using a basic tissue engineering approach. To address this problem, some researchers have reported that culturing cells on scaffolds with anisotropic structures would induce anisotropic remodeling of cartilaginous tissue [11,12]. Mechanical stimulation is another approach to regenerating anisotropic tissue. Lee et al. cultured scaffold-free engineered tissue with tensile stimulation to regenerate anisotropic human neocartilage [13]. Anisotropic mechanical properties were also observed in the fibroblast-seeded gels subjected to mechanical stretching [14]. Our research group also reported that cyclic compression improved the stiffness and mechanical anisotropy of the regenerated cartilaginous tissue [15]. From previous studies, anisotropic cellular organization was required to simulate “native” biological tissue. In this study, we focused on the direct control of cellular organization in hydrogels to regenerate 3-D anisotropic cartilaginous tissue. The random encapsulation of chondrocytes within hydrogels has been widely used for the in vitro culture of articular chondrocytes [16,17,18,19,20]. However, the control of cellular organization in hydrogels remains difficult. While articular cartilage has been one of the first tissues to be successfully treated with tissue engineering therapy, regenerating the anisotropic architecture and biomechanical properties of articular cartilage remains a challenging problem.

Recently, a novel micro-particle patterning technique was developed that utilizes DEP forces to manipulate living cells [21,22,23,24,25,26,27]. Albrecht et al. reported the encapsulation of living cell arrays using dielectrophoresis and photo-cross-linked hydrogels [23,24,25]. In our previous studies, we reported cell manipulation and separation systems [28,29,30] and a cell-patterning technology in hydrogels using dielectrophoresis [31]. However, there have not been any studies on remodeling the mechanical anisotropic tissue based on controlled cellular organization by dielectrophoresis.

This study aimed to perform DEP cell patterning in a hydrogel to modulate cellular organization, proliferation, and extracellular matrix synthesis to regenerate mechanically anisotropic cartilaginous tissue. First, the dielectrophoretic properties of primary articular chondrocytes were evaluated to determine the optimal conditions for manipulating the cells in agarose gel. Based on the measured dielectrophoretic properties, the chondrocytes were patterned in an anisotropic alignment inside the agarose gel using negative-DEP forces. Mechanical tensile tests were performed to characterize the mechanical anisotropy of the chondrocyte-aligned hydrogel constructs. 

## 2. Materials and Methods

### 2.1. Chondrocyte Isolation for Dielectrophoresis

Articular cartilage was harvested from articular joints of 3–6-week-old calves from a local abattoir, and chondrocytes were isolated from cartilage explants by enzymatic digestion [15]. Briefly, cartilage explants were excised from the humeral head and minced into small pieces, which were then shaken gently in 0.2% collagenase type II digested with Dulbecco’s modified Eagle’s medium/Ham’s F12 (DMEM/F12) supplemented with 5% fetal bovine serum (FBS) and antibiotics–antimycotics for 8 h at 37 °C. The cartilage-digested solution was filtered through a 70 μm nylon mesh filter to remove debris. The cells were isolated from the digest by centrifugation for 5 min and rinsed twice with phosphate-buffered saline (PBS) containing antibiotics–antimycotics. Finally, after centrifugation for 5 min, the cells were resuspended in a low-conductivity osmotically balanced buffer (LC buffer: 10 mM HEPES, 0.1 mM CaCl_2_, and 59 mM D-glucose in sucrose solution [28,29,30,31]) for dielectrophoresis experiments. The total cell number was counted using a hemocytometer to adjust the cell concentration prior to the experiments.

### 2.2. Dielectrophoresis Chamber for Living Cells

**Figure 1:**
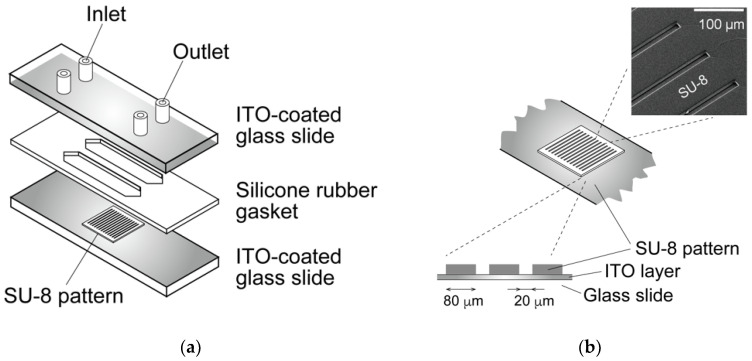
Schematic of the dielectrophoretic chamber. (**a**) The chamber was constructed by sandwiching a silicone rubber gasket between two glass slides coated with indium tin oxide (ITO). (**b**) The bottom slide was partially insulated by an SU-8 pattern to fabricate a parallel-line electrode array.

**Figure 2:**
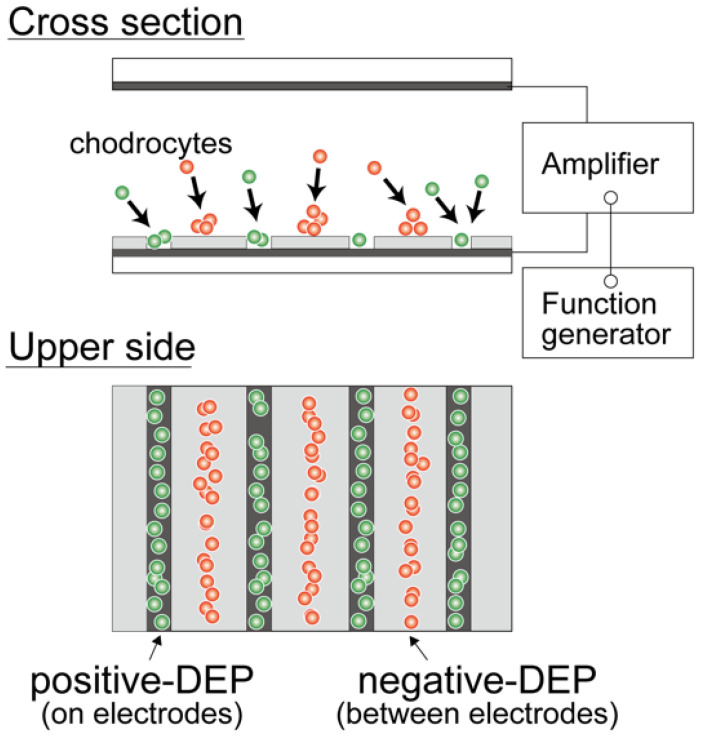
Dielectrophoresis of living cells. Cells were moved to a region with a low electric field (between the electrodes) or high electric field (on the electrodes).

A dielectrophoresis chamber for cellular analysis and accumulation was developed to establish a nonuniform electric field in a rectangular volume. The chamber was constructed by sandwiching a 500 μm silicon rubber gasket between two glass slides coated with a conductive material, indium tin oxide (ITO) (Figure 1a). The bottom-side glass was partially coated with a 1 μm film of SU-8 photoresist to insulate specific areas of the conductive surface and fabricate a parallel line electrode array with a width of 20 μm; the lines were spaced 80 μm apart (Figure 1b). A sine-wave AC voltage was applied between the upper and bottom ITO-coated glass slides to generate a non-uniform electric field. DEP is a phenomenon that occurs under an applied non-uniform electric field, inducing dipoles within a polarized cell in a buffer solution. The cell, in a non-uniform electric field, can be moved by DEP forces toward high or low electric field regions, depending on the relative electric property of cells, which is related to the cell type and function [21,28]. A high electric field is localized at the line-shaped electrodes, and cells are localized to the high or low electric field region due to positive- or negative-dielectric forces depending on their electric properties (Figure 2). The AC voltage was applied between the parallel-line electrode-fabricated glass slide and upper ITO-coated glass slide using a function generator (WF1944B, NF Corp., Yokohama, Japan) and an amplifier (BA4850, NF Corp., Yokohama, Japan). The applied voltage was monitored using an oscilloscope (TDS1001B, Tektronix, Beaverton, OR, USA) connected in parallel. The movement of cells within the DEP chamber was observed using a phase-contrast microscope (CKX41, Olympus, Tokyo, Japan) with a digital video camera. The dielectrophoretic chamber was sterilized with 70% ethanol followed by rinses with a low-conductivity (LC) buffer containing 1% Pluronic F108 (Sigma-Aldrich, St. Louis, MO, USA) prior to the dielectrophoresis experiments. Pluronic 108 was used to avoid cell adhesion to the glass slides after dielectrophoresis experiments. In our previous study [31], it was confirmed that cell viability and function would be maintained under the dielectrophoretic conditions of this study.

### 2.3. Dielectrophoretic Characterization of Chondrocytes

**Figure 3:**
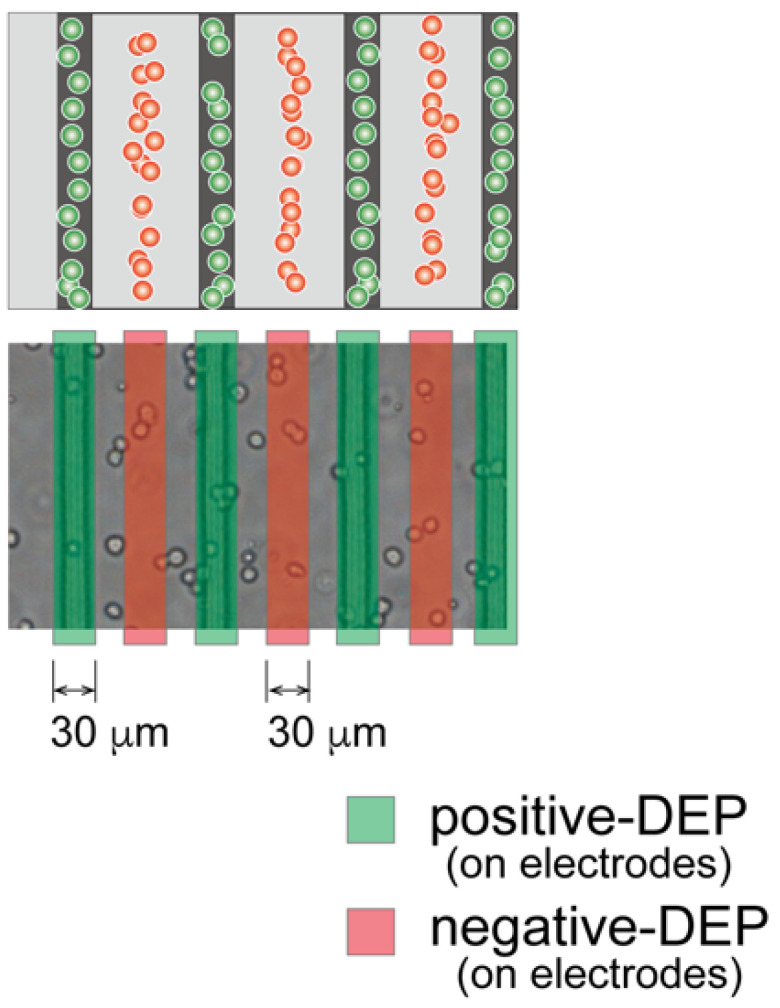
Evaluation of the dielectrophoretic property of chondrocytes. After dielectrophoresis, cells in red regions demonstrated a negative-DEP response, whereas those in green regions demonstrated positive-DEP response.

For the DEP characterization, chondrocyte suspension in LC buffer was injected into the DEP chamber and subjected to an AC voltage for 180 s after injection. The magnitude of the imposed AC voltage was 10 V_p-p_ with a frequency varying from 10 kHz to 1 MHz. The behavior of chondrocytes was observed using a digital camera on the microscope, and microphotographs were captured 180 s after each AC voltage frequency was imposed. The number of cells on the electrodes (positive-DEP) and between the electrodes (negative-DEP) were counted in each captured image. The ratios of cells indicating positive- and negative-DEPs in the chamber were calculated to evaluate the crossover frequency. The dielectrophoretic property of a cell (indicating p-DEP or n-DEP) was assessed based on the region where the cell moved (Figure 3). The frequency dependency of the DEP property was evaluated by the positive-DEP ratio *R*_p_ and the negative-DEP ratio *R*_n_, calculated as follows:

*R*_p_ = *N*_P_/(*N*_P_ + *N*_n_), *R*_n_ = *N*_n_/(*N*_P_ + *N*_n_), (1)

where *N*_P_ and *N*_n_ are the numbers of cells indicating positive-DEP and negative-DEP, respectively. To evaluate the dielectrophoretic property of cells, the dielectrophoresis parameter was defined as *R*_p_–*R*_n_.

### 2.4. Dielectrophoretic Cell Accumulation in Agarose Gel to Fabricate Cell-Aligned Three-Dimensional Cultures

Based on dielectrophoretic characterization, chondrocytes were patterned in agarose gel via the negative-DEP force. Chondrocyte-suspended agarose solutions were prepared as previously described [15,32,33,34,35]. The isolated chondrocytes were suspended in the LC buffer and mixed in a 1:1 ratio with 3% low-melting agarose (A2576; Sigma-Aldrich, St. Louis, MO, USA) in the LC buffer to make a 1.5% agarose solution with a cell density of 1.0 × 10^7^ cells/mL. For cell accumulation, the chondrocyte/agarose solution was injected into the chamber. A sine-wave AC voltage of 15 V_p-p_ and 10 kHz was applied for 15 min to cause rapid cell localization in the region of low electric field (negative-DEP). After chondrocyte accumulation, the chondrocyte/agarose solution in the chamber was cooled at 4 °C for 20 min to obtain a gelation agarose solution. 

**Figure 4:**
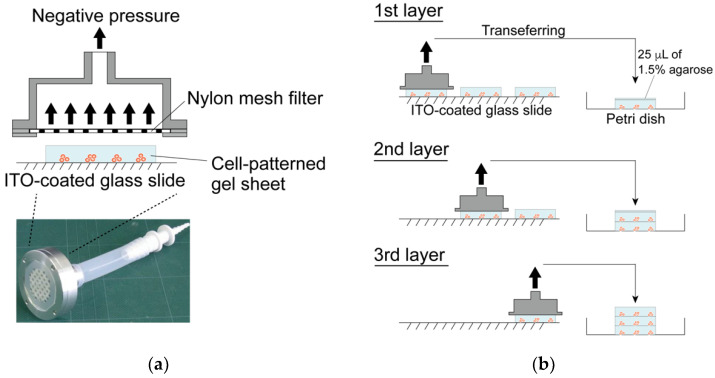
Fabrication of the layered gel-sheet construct. (**a**) Gel sheet transferring device using negative air pressure and (**b**) layering process of the cell-patterned agarose gel sheets.

Three agarose gel sheets containing patterned-chondrocyte arrays were stacked to form a 3-D culture sample for mechanical anisotropy evaluation. The gel sheets in the DEP chamber were transferred to a Petri dish using a custom-made gel sheet holder using negative pressure suction. The gel sheet holder was constructed from a nylon mesh sheet and stainless-steel parts. The gel sheets were held by adsorbing the sheet onto the nylon mesh through aspiration from a syringe (Figure 4). After the first sheet was transferred to the dish, the second and third gel sheets were collected and stacked onto the already transferred gel sheet using the same procedure. Then, a 25 μL 1.5% agarose solution was dropped between the sheets as adhesive glue. The position of each gel sheet was aligned manually under a stereomicroscope. The stacked gel-sheet construct was shaped into a thin plate 5 × 15 mm^2^ with a thickness of 1.5 mm (500 μm × 3 sheets) for cell culture experiments. To evaluate mechanical anisotropy, three types of specimens were fabricated; the cells were patterned parallel or perpendicular to the longitudinal direction of the gel sheet and were homogeneously dispersed to prepare three experimental groups (i.e., parallel, perpendicular, and homogeneously dispersed groups). 

The chondrocyte-accumulated agarose gel constructs were cultured for up to 21 days in DMEM/F12 containing 20% FBS and 50 μg/mL ascorbic acid. The cultures were maintained in a humidified tissue culture incubator at 37 °C and 5% CO_2_.

### 2.5. Biomechanical Characterization of the Regenerated Tissue

**Figure 5:**
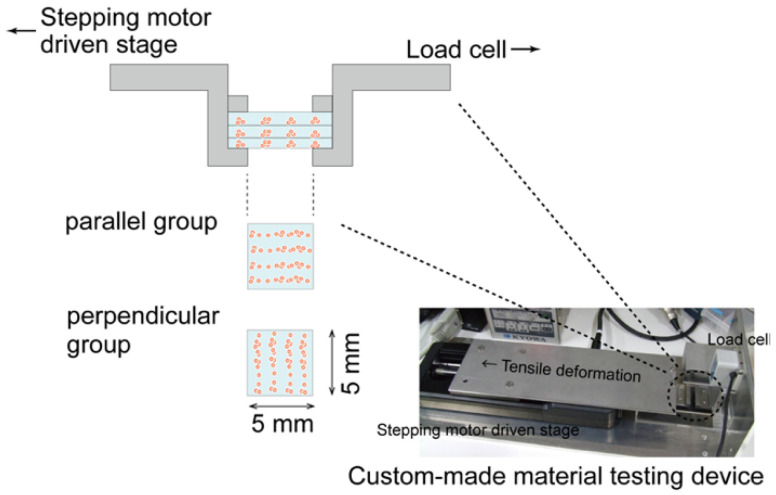
Evaluation of the mechanical properties of layered gel-sheet constructs. Specimens were stretched parallel or perpendicular to the cellular alignment in the gel-sheet construct.

**Figure 6:**
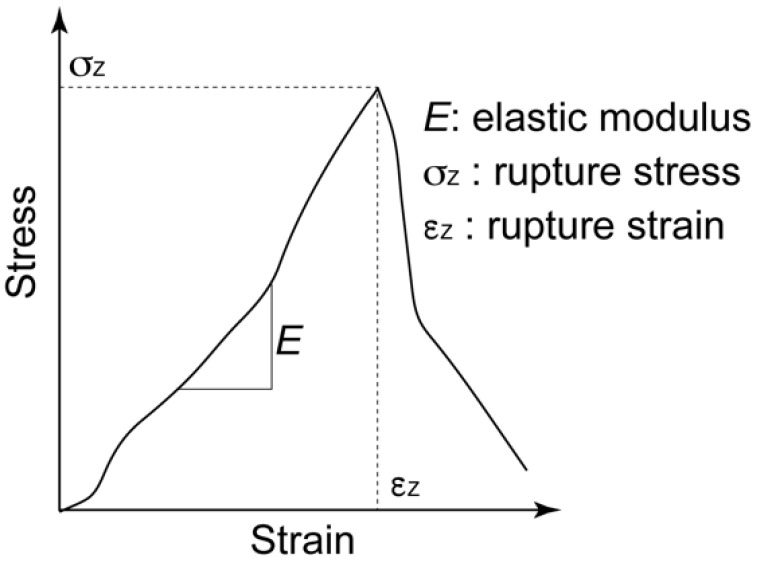
Definition of elastic modulus (*E*), rupture stress (σ_z_), and rupture strain for the stress–strain curve of the gel-sheet laminated construct.

After 7, 14, and 21 days of culturing, the mechanical properties of the cultured construct were evaluated using a custom-made material testing device. The material testing device consisted of a load cell (Kyowa Electronic Instruments, Tokyo, Japan), stepping motor driven stage (Sigma-Koki, Saitama, Japan), and stainless-steel grips for the gel sheet (Figure 5). The stacked-gel-sheet construct was gripped to expose a 5 × 5 mm^2^ area, and tensile deformation was applied. The specimen was stretched at a strain rate of 0.01/s up to a strain of 0.4. The stress and strain were calculated using the measured displacement and load values. The elastic modulus, rupture stress, and rupture strain were measured from the stress–strain curve. Regarding the analysis of the hydrogel strain–stress curve, the stress at the point of maximum stress was defined as the rupture stress, and the strain at that point was defined as the rupture strain (Figure 6).

### 2.6. Cell Proliferation, Viability, and Biochemical Composition of the Regenerated Tissue

The biochemical properties of the cultured constructs were evaluated after 7, 14, and 21 days of culturing. For histological analysis, the cultured samples were fixed in a 4% paraformaldehyde solution at 4 °C overnight, followed by staining with Safranin-O to evaluate the sGAG distribution. For biochemical characterization, the samples were digested in papain (125 μg/mL in PBS) at 60 °C for 6 h. Each digested lysate was independently assayed for GAG content using a dimethylmethylene blue assay [34,36] and for DNA content using a fluorometric DNA assay with Hoechst 33258 [37]. The cell number in the cultured construct was calculated from the total DNA content divided by the cellular DNA content (7.7 pg). The viability of the chondrocytes in the agarose gel was assessed using live/dead staining. The cultured constructs were incubated in 1 mL of DMEM containing 1 μg calcein-AM (Dojindo, Japan) and 2 μg propidium iodide (PI; Dojindo, Kumamoto, Japan) at 37 °C for 30 min. Fluorescent images were captured using a fluorescence microscope (CKX41, Olympus, Tokyo, Japan). Dead cells appeared red, while viable cells were green. 

### 2.7. Statistical Analysis

Most of the data were representative of three individual experiments with similar results. The statistical significance of the experimental data was evaluated using the Tukey–Kramer method. Statistical significance was set to *p* < 0.05. 

## 3. Results

### 3.1. Dielectrophoretic Property of Primary Chondrocytes

**Figure 7:**
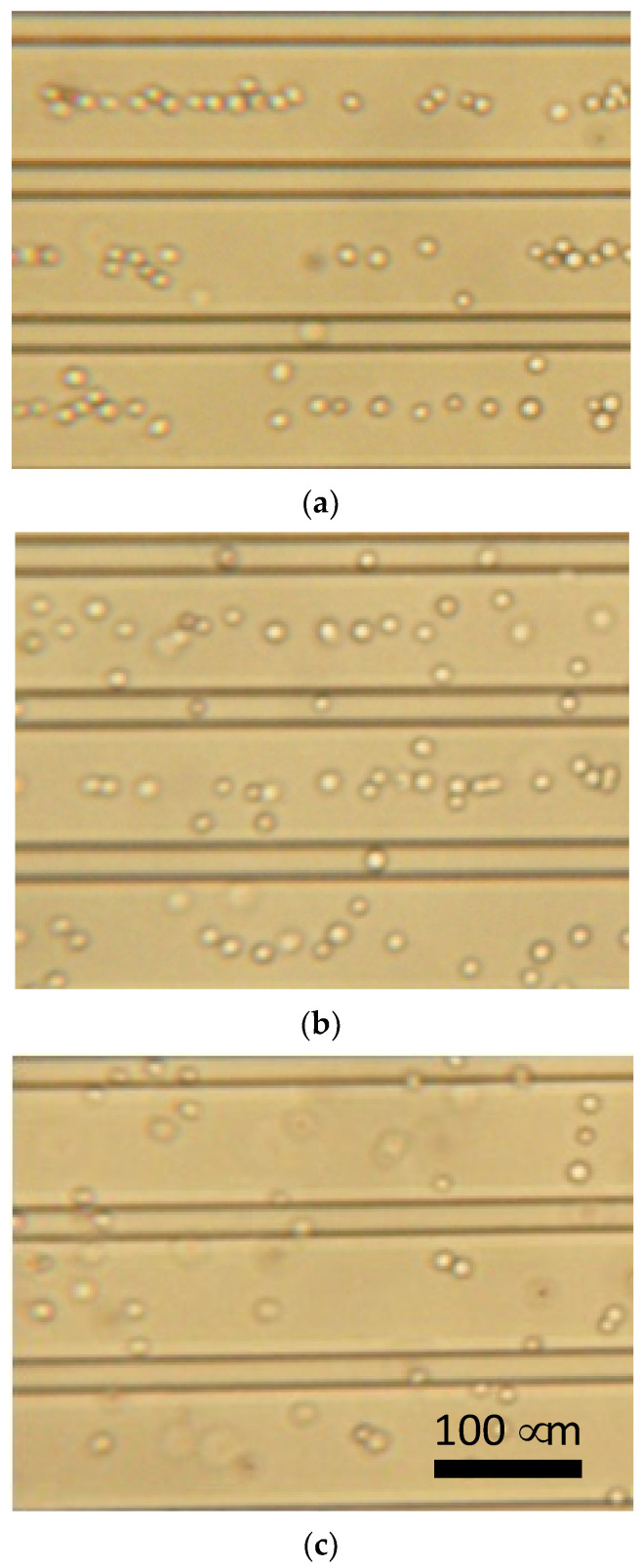
Dielectrophoresis of chondrocytes using parallel line-electrode array. (**a**) 10 kHz, (**b**) 100 kHz, and (**c**) 500 kHz of AC voltage.

The primary chondrocytes changed the DEP responses depending on the frequency of the AC voltage. Figure 7 shows photomicrographs of chondrocytes under DEP at different frequencies. The chondrocytes showed a negative-DEP at 10 kHz, whereas a positive-DEP was observed at 500 kHz. Moreover, the chondrocytes showed both negative- and positive-DEPs at frequencies of approximately 100 kHz. 

**Figure 8:**
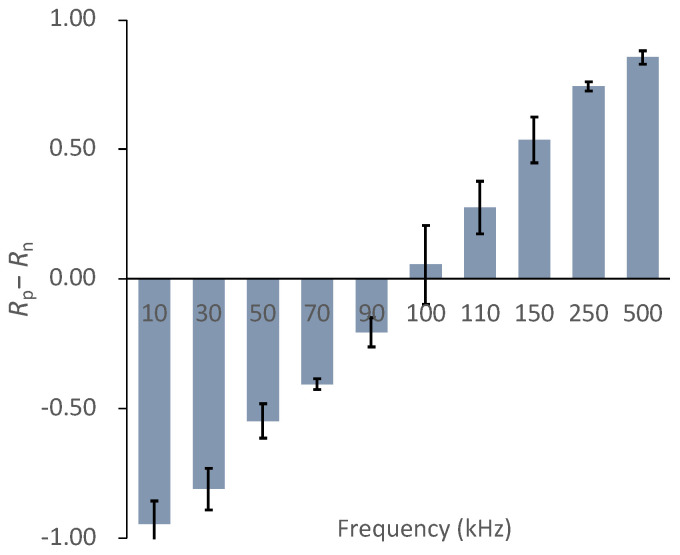
DEP frequency dependency parameter, *R*_p_−*R*_n_, of chondrocytes. Mean ± S.D., *n* = 5.

To evaluate the DEP frequency dependency of the cells, we defined *R*_p_–*R*_n_ as the frequency-dependent parameter. Figure 8 shows the ratio of chondrocytes expressing positive- and negative-DEP responses (*R*_p_–*R*_n_). The chondrocytes switched from positive- to negative-DEPs between 90 and 110 kHz. The result indicates that the crossover frequency of the primary chondrocyte DEPs was approximately 100 kHz (Figure 8).

### 3.2. Tissue Reconstruction in Chondrocyte-Organized Agarose Gel Constructs

**Figure 9:**
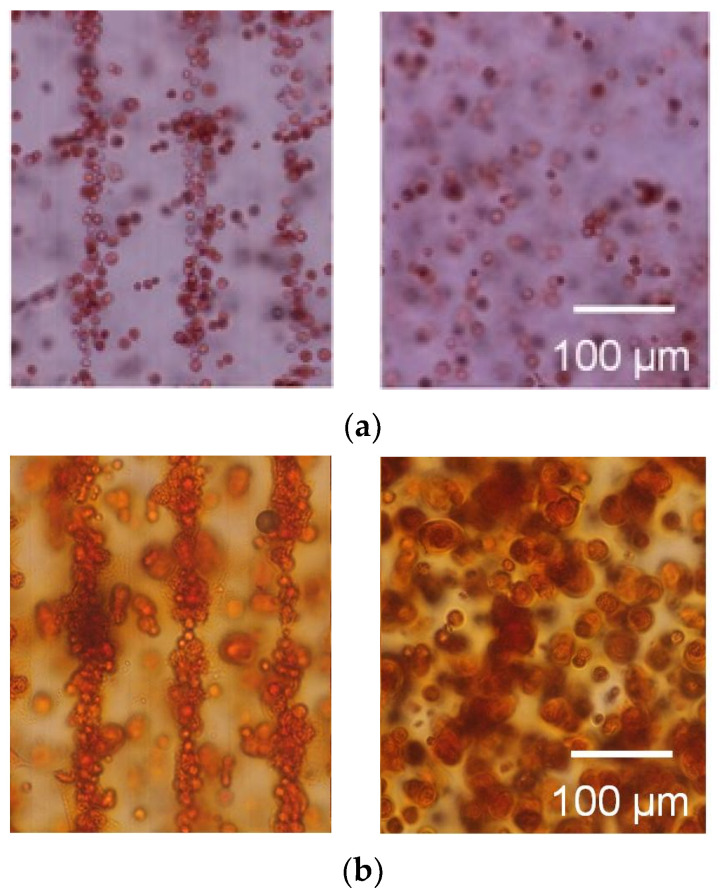

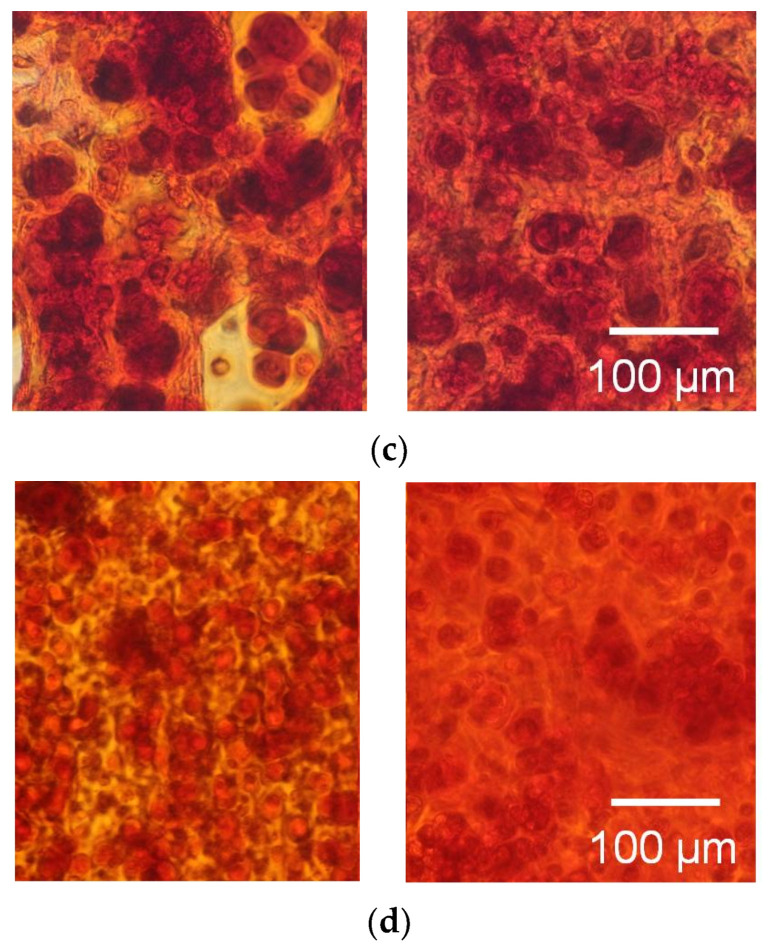
Safranin-O staining of the agarose gel-sheet constructs cultured for (**a**) 0, (**b**) 7, (**c**) 14, and (**d**) 21 days. Left: cell-patterned specimens; right: homogeneously cell-dispersed specimens.

**Figure 10:**
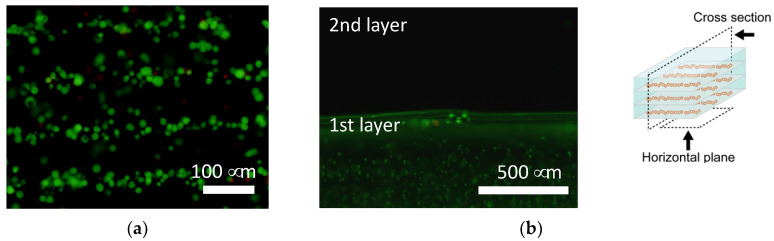
Calcein-AM staining of the agarose gel-sheet constructs cultured for 7 days. Fluorescence images captured from (**a**) the horizontal plane and (**b**) the cross section.

The cells were patterned in an agarose gel sheet based on the measured DEP properties of chondrocytes. Following the cell patterning in gel sheets and lamination of the cell-patterned gel sheets, the cell arrays were retained to form anisotropic tissue during the culturing period. From the safranin-O staining of the gel-sheet layered construct, cartilaginous tissue containing sGAG was reconstructed along the patterned cell array, whereas the chondrocytes seeded homogeneously in agarose gels secreted and reconstructed homogeneous tissue (Figure 9). The cartilaginous tissue in the cultured agarose gel expanded with increasing culture time. Each reconstructed tissue along the cell array was connected to adjacent tissues over a seven day culturing period. Cell viability was maintained in the gel-sheet layered constructs, and in the homogeneously chondrocyte-seeded agarose gel (Figure 10). 

**Figure 11:**
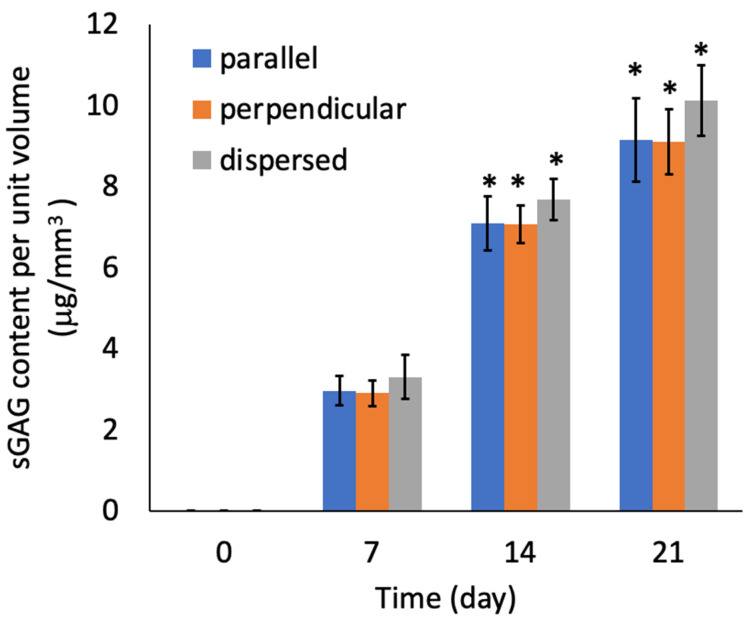
Sulfated GAG content of the agarose gel-sheet constructs cultured for 21 days. Mean +/− S.D., *n* = 5. * indicates a significant difference in each value compared to day 7, *p* < 0.05.

**Figure 12:**
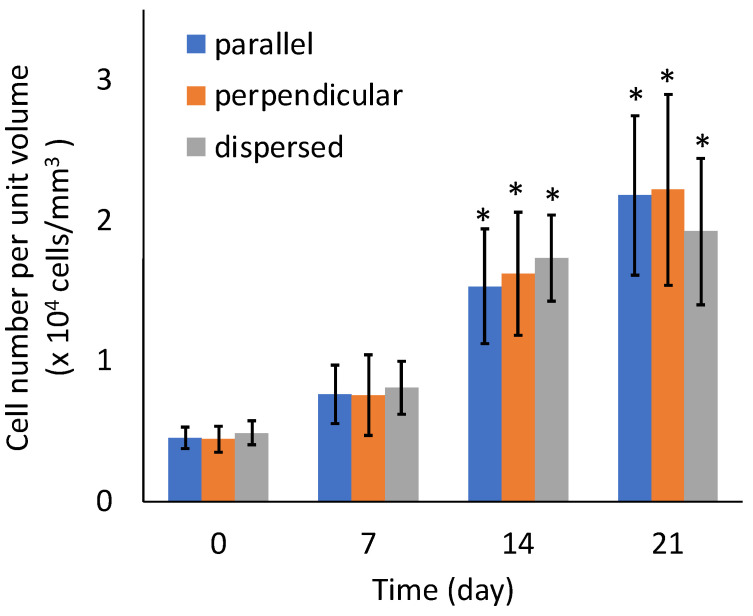
Cell number of the agarose gel-sheet constructs cultured for 21 days. Mean +/− S.D., *n* = 5. * indicates a significant difference in each value compared to day 0, *p* < 0.05.

There were no significant differences in the sGAG contents of the specimens from the three experimental groups (Figure 11). There were also no significant differences in the number of cells among the three experimental groups (Figure 12). The sGAG contents and cell numbers of specimens in all experimental groups increased with increasing culturing time.

### 3.3. Mechanical Anisotropy of the Regenerated Tissue

**Figure 13:**
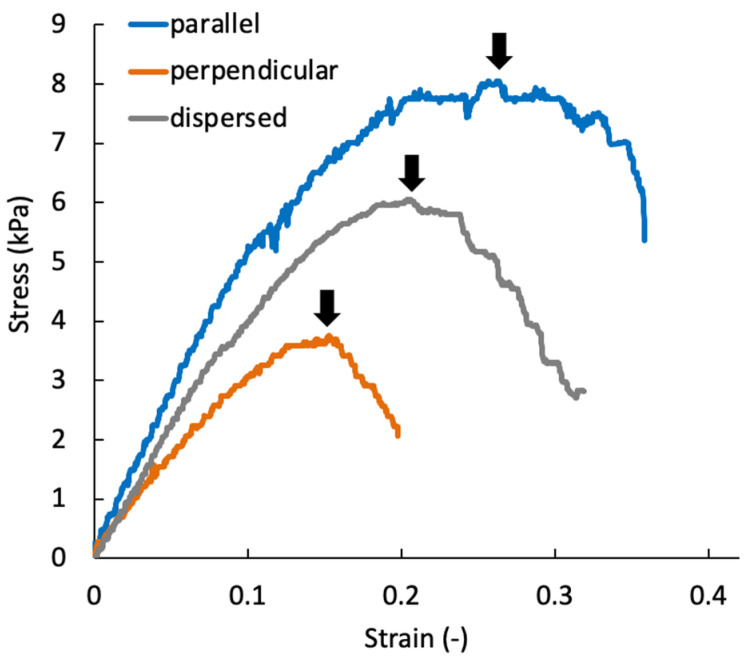
Representative stress–strain diagram of the agarose gel sheet-laminated construct containing chondrocytes cultured for 21 days. Black arrows indicate the rupture point of each specimen.

**Figure 14:**
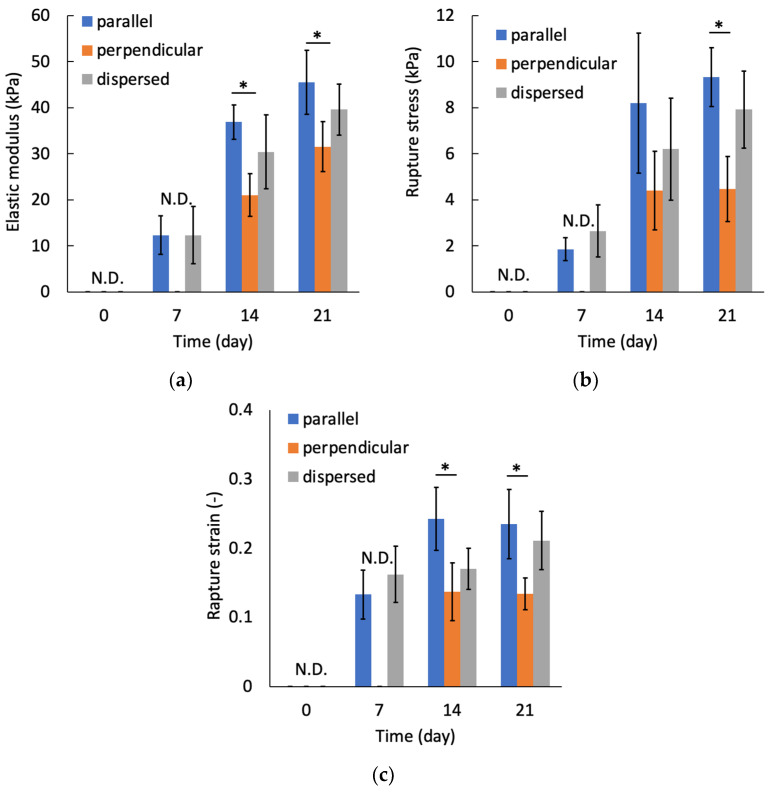
Relationships between culture periods and (**a**) elastic modulus, (**b**) rupture stress, and (**c**) rupture strain. Mean +/− S.D., *n* = 4. * indicates a significant difference between parallel and perpendicular groups at each time point, *p* < 0.05.

At day 0, the tensile mechanical tests could not be performed because the stiffness of specimens was not sufficient to handle them. The tensile test for perpendicular specimens at day seven also could not be performed. Figure 13 shows a typical stress–strain diagram for each sample group on day 21. The results of tensile tests show that the specimens were fractured at tensile strains smaller than 0.4 for all specimen groups. In this study, the elastic modulus was calculated by linear approximation in the region of strain 0.05–0.1. The elastic modulus increased with increasing culturing time for all sample groups (Figure 14a). On days 14 and 21, the elastic modulus of the parallel group, in which chondrocytes were patterned parallel to the tensile direction, was significantly larger than that of the perpendicular group. The parallel group also showed significantly larger values of rupture stress and strain compared with the perpendicular group (Figure 14b,c). 

## 4. Discussion

In this study, to regenerate cartilaginous tissue with mechanical anisotropy simulating that of “native” articular cartilage, we established a 3-D culture method to accumulate chondrocytes in a parallel-line pattern inside agarose gels by negative dielectrophoresis. There are many reports on the three-dimensional culture of calf chondrocytes using agarose gels as scaffold material, which state that cartilaginous tissue was reconstructed inside the gel [16,17,18,19,20]. In our study, chondrocytes synthesized an extracellular matrix inside the agarose gel and reconstructed cartilaginous tissue along the parallel-line pattern of accumulated cells. There was no significant difference in the sGAG content among the experimental groups where the chondrocytes were patterned, and the group where the chondrocytes were uniformly distributed inside the gel during the 21-day culturing period. This result indicated that the manipulation of cells by dielectrophoresis and cell accumulation did not affect the cell viability or ability to synthesize extracellular matrix.

The elastic modulus of cultured constructs increased with an increase in the culturing period. This increase in elastic modulus showed a similar trend to that reported in previous studies [17,18]. Furthermore, mechanical anisotropy of the regenerated tissue was observed on days 14 and 21. The elastic modulus of the cultured construct with chondrocytes patterned in the same direction as the tensile direction was significantly higher than that of the sample with perpendicularly patterned chondrocytes. The sGAG content in the cultured construct was not significantly different among the experimental groups. However, it is thought that the extracellular matrix reconstructed around the patterned cell arrays became denser and that the tissue structure became more oriented, resulting in a higher stiffness in the direction of cell patterning. In the cultured specimens from the parallel group, the regenerated tissue was not anisotropic in geometry because the tissue was formed to be connected to adjacent cell arrays, whereas significant mechanical anisotropy was observed on day 21. It was assumed that the dense extracellular matrix with orientation along each cell array, which was formed during the early 14-day culture, was still contained in the 21-day cultured specimens. In this study, the gel sheets containing cell-array were stacked to reconstruct the anisotropic biological tissue. To achieve the higher functionality of regenerated tissue, lamination of the cell sheets in an aligned state [38] will be required. Our dielectrophoresis and stacking method will be applicable to align the cell sheets as a pattern and to stack the sheets.

Inside the structure of native articular cartilage, cells and collagen fiber networks are oriented parallel to the articulating surface in the top tissue layer, randomly in the middle layer, and vertically in the deep layer [1,10]. This complex structure enables articular cartilage tissue to withstand shear, tensile, and compressive deformations caused by motion, such as daily walking. To reconstruct this complex structure of articular cartilage, a three-dimensional structure of electrode patterns regarding dielectrophoresis and a more complex assembly technique regarding the gel sheets are required. It has been shown that the mechanical anisotropy of biological tissues represented by articular cartilage can be reconstructed using anisotropic scaffold materials or mechanical stimulation [11,12,13,39]. However, it is difficult to replicate the orientation of chondrocytes in vivo using these methods. The dielectrophoretic cell patterning technique inside hydrogels proposed in this study can control the cellular orientation in the scaffold material. Furthermore, this cell accumulation method would enable the reconstruction of tissue structures similar to those of “native” articular cartilage at a cellular organization level. 

## 5. Conclusions

In this study, chondrocytes were accumulated to form parallel-line cell clusters in agarose gels via negative-DEP forces. The tensile strength of the chondrocyte-accumulated agarose gel construct, sGAG content, and cell number count increased with increasing culturing time. The stiffness of the constructs in which chondrocytes were patterned in the same direction as the tensile direction was significantly greater than that of the samples with perpendicularly patterned chondrocytes. Finally, the DEP cell-accumulating methodology inside a hydrogel could become a promising approach for regenerating mechanically anisotropic tissues in cartilaginous tissue reconstruction.
